# The Effects of Corticosteroids on the Respiratory Microbiome: A Systematic Review

**DOI:** 10.3389/fmed.2021.588584

**Published:** 2021-03-10

**Authors:** Julia E. Hartmann, Werner C. Albrich, Marija Dmitrijeva, Christian R. Kahlert

**Affiliations:** ^1^Division of Infectious Diseases/Hospital Epidemiology, Kantonsspital St. Gallen, St.Gallen, Switzerland; ^2^Department of Molecular Life Sciences, University of Zurich, Zurich, Switzerland; ^3^Swiss Institute of Bioinformatics, Zurich, Switzerland; ^4^Division of Infectious Diseases/Hospital Epidemiology, Children's Hospital of Eastern Switzerland, St. Gallen, Switzerland

**Keywords:** corticosteroid, respiratory microbiome, airway disease, systematic review, diversity

## Abstract

**Background:** Since its discovery, the respiratory microbiome has been implicated in the pathogenesis of multiple pulmonary diseases. Even though corticosteroid treatments are widely prescribed for pulmonary diseases, their effects on the respiratory microbiome are still poorly understood. This systematic review summarizes the current understanding of the effects of corticosteroids on the microbiome of the airways.

**Research Question:** How does treatment with corticosteroids impact the respiratory microbiome?

**Study Design and Methods:** According to the PRISMA guidelines, Embase, Medline, and the Cochrane Central Register of Controlled Trials (CENTRAL) databases were systematically searched for all observational or randomized-controlled studies comparing the microbiome parameters of patients receiving corticosteroids to those of controls. The primary outcomes of interest were changes in the diversity, composition and total burden of the respiratory microbiome as assessed by culture-independent molecular methods.

**Results:** Out of 1,943 identified reports, five studies could be included: two on patients with asthma, two on patients with chronic obstructive pulmonary disease and one on patients with chronic rhinosinusitis. The studies were highly heterogeneous with regards to the methods used and the populations investigated. Microbiome diversity increased with corticosteroids at least transiently in three studies and decreased in one study. The effects of corticosteroids on the composition of the respiratory microbiome were significant but without a clear shared direction. A significant increase in microbial burden after corticosteroids was seen in one study.

**Interpretation:** Data on the effect of corticosteroids on the respiratory microbiome are still limited, with considerable heterogeneity between studies. However, available data suggest that corticosteroid treatment may have significant effects on the composition and possibly the diversity of the respiratory microbiome. Further research is needed to better understand the influence of corticosteroids on the respiratory microbiome and thus better target its widespread therapeutic use.

## Introduction

**Figure 1:**
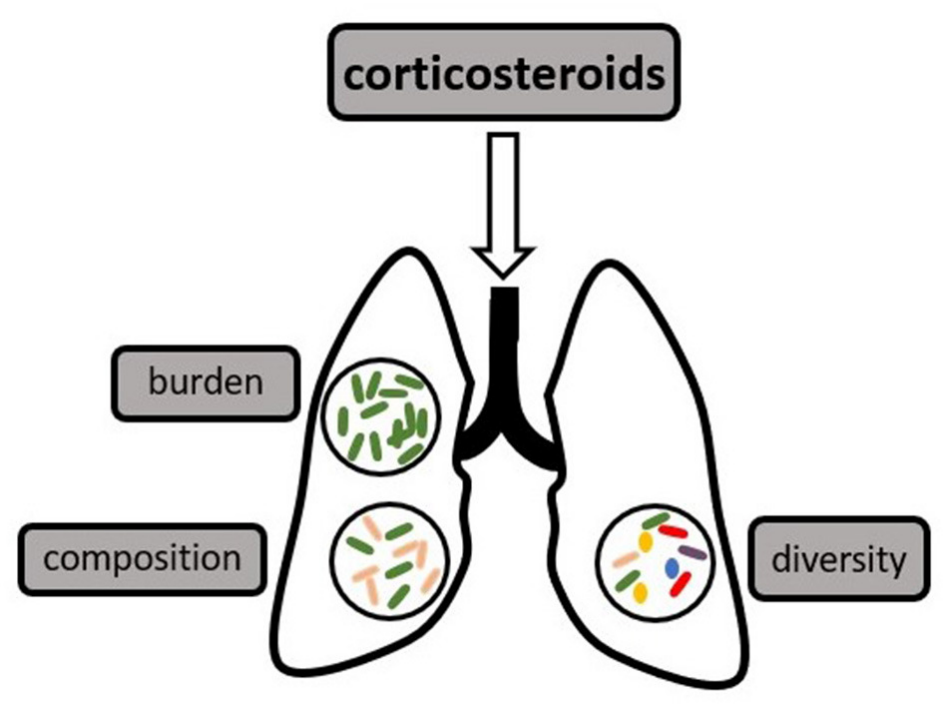
Parameters of a microbiome: burden, composition, and diversity.

The human microbiome has a high inter-individual variation both on a taxonomic and a functional level and has been shown to have a significant impact on human health and disease (1, 2). The most densely populated and best researched habitat is the gut microbiome, which was found to provide essential nutrients for the host, enhance local defenses against enteral pathogens, and shape systemic immunity (1, 3, 4). Dysbiosis of the gut microbiome has been linked to multiple disease states, including inflammatory bowel disease and critical illness (5, 6). Measures of diversity are possibly the most well-known parameter describing a microbiome (7), however, the composition (or relative abundance of certain phyla or taxa), as well as the total microbial burden are also relevant characteristics (1, 8–10), as demonstrated in Figure 1.

The gut-lung axis is a recently coined term to describe the increasingly appreciated contribution of the gut microbiota to the immunity in the lung (11) and to the pathogenesis of a number of lung diseases (e.g., allergic asthma after antibiotic treatment in childhood) (12). Gut microbiota-depleted mice were shown to be more susceptible to pneumococcal pneumonia than controls with an intact gut microbiome, as demonstrated by a significantly higher bacterial load, more organ damage, and a higher mortality rate. Importantly, fecal microbiota transfer (FMT) restored the bacterial clearance of the lung (13). Similarly, another murine study found that commensal gut microbiota drives the interferon signature, which is implicated in antiviral defense, specifically that of the lung. Antibiotic treatment-associated changes in gut microbiota resulted in a blunted interferon signature and increased influenza virus replication in infection, all of which could be reversed by FMT (14). A randomized-controlled study of children suffering from acute lung injury found significantly lower levels of inflammatory factors and less small bronchial obstruction in children treated with probiotics when compared to placebo-treated controls, whose inflammatory factors did not drop as rapidly and whose pulmonary function was still limited after 10 days (15). Although some aspects of the interaction between the intestinal- and the lung microbiome have been described, the complex cross—talk, the causality between lung diseases and gut microbiota is still underexplored (16).

Just in the last decade it was discovered that the healthy lung itself also possesses a small but diverse microbiome of its own, which are generally indistinguishable in their composition from upper airway microbiota (17–19). Currently, the lung microbiome is still poorly understood and its study poses multiple methodological challenges, e.g., the necessity of invasive sampling and the concurrent risk of contaminating samples with upper respiratory tract microbiota (20). Despite these challenges, multiple studies have provided insights into the lung microbiome with respect to its diversity, composition (i.e., relative abundances of certain phyla or taxa), and microbial burden particularly in connection with chronic lung diseases.

An increasing number of lung diseases (asthma, lower respiratory tract infections) have been associated with changes to the lung microbiome during childhood, for example, through antibiotic treatment (21). Two studies found significantly higher percentages of Proteobacteria in asthma patients when compared to healthy controls (19, 22). Airway microbiota composition from patients with respiratory diseases differed significantly from healthy controls (22, 23). In ventilated patients colonized with *Pseudomonas aeruginosa*, a decrease in the diversity of the lung microbiome under antibiotic treatment was highly associated with the development of pneumonia (24). Further supporting this hypothesis, a study of HIV-infected patients with acute pneumonia observed an inverse correlation between the richness and phylogenetic diversity of the lung microbiome and the bacterial burden during pneumonia (10).

However, although treatments containing corticosteroids (CS) are frequently prescribed to patients with chronic lung diseases, little is known about their effect on the respiratory microbiome. In order to make better informed clinical decisions, a clearer understanding of the impacts of CS on the bacterial communities of the airways is essential.

We thus systematically reviewed and summarized studies using culture-independent methods to investigate changes in the airway microbiome in patients receiving CS treatment for respiratory diseases when compared to controls. The primary outcomes assessed were changes in microbial diversity, composition, and total burden.

## Methods

This systematic review was performed according to PRISMA guidelines (25). The protocol including the research question including defined inclusion criteria for populations, interventions, comparators, outcomes, and study designs (PICOS) was published on the Prospero database (ID: CRD42019137012). The **population** of interest included any patients receiving CS treatment for respiratory tract diseases without age restriction. Eligible **interventions** were applications of steroids that were systemic (e.g., oral) and topical applications to the respiratory system (this includes inhaled and topical nasal applications). Topical applications to organ systems other than the respiratory tract were excluded. Eligible **comparators** were healthy controls or patients on standard of care (SOC), placebo-treatment, or not undergoing treatment. The definition of SOC varied depending on the underlying condition. For patients with asthma or stable chronic obstructive pulmonary disease (COPD), this mainly included bronchodilators. For patients with COPD exacerbations, SOC could be antibiotic treatment.

The **outcomes** assessed during this systematic review were microbiome composition, diversity, and total burden in the respiratory tract, and changes in these parameters after exposure to CS as determined by culture-independent molecular methods. The microbiome **composition** was expressed through the relative abundances of different phyla or taxa in a sample. The **diversity** measure of interest for this review was α-diversity (i.e., diversity within a sample), determined using the relative inverse Simpson index, Shannon index or Faith's phylogenetic diversity. Diversity indices considered both community richness and evenness. The total microbial **burden** was related to the number of bacteria present (16S rRNA copy number frequently employed as a proxy). Randomized trials and observational (case-control and cohort) studies were considered eligible **study designs**.

The electronic databases Medline, Embase (both via Ovid), and the Cochrane Library (Cochrane Database of Systematic Reviews, Cochrane Central Register of Controlled Trials (CENTRAL), Cochrane Methodology Register) were systematically searched in June 2019. The search strategies included database-specific subject headings and free text synonyms for respiratory tract, CS, and microbial analyses. The detailed search strategies can be found in the Supplementary Material. As a supplementary search technique, the bibliographic references and citations of all included articles indexed in Scopus or the Web of Science were screened in order to identify possible additional studies that escaped our electronic database searches. There were no language or publication date restrictions. The risk of bias assessment was performed with two different tools, ROB 2.0 for RCTs and the Newcastle Ottawa Scale (NOS) for cohort studies. As there is no standardized established interpretation of the NOS, we applied the most commonly used method applied in the available literature, in which a “good” quality score requires 3 or 4 stars in selection, 1 or 2 stars in comparability, and 2 or 3 stars in outcomes. A “fair” quality score requires 2 stars in selection, 1 or 2 stars in comparability, and 2 or 3 stars in outcomes. A “poor” quality score reflects 0 or 1 star(s) in selection, or 0 stars in comparability, or 0 or 1 star(s) in outcomes.

## Results

### Strict Application of the Protocol Results in Only Five Studies That Can Be Included

**Figure 2:**
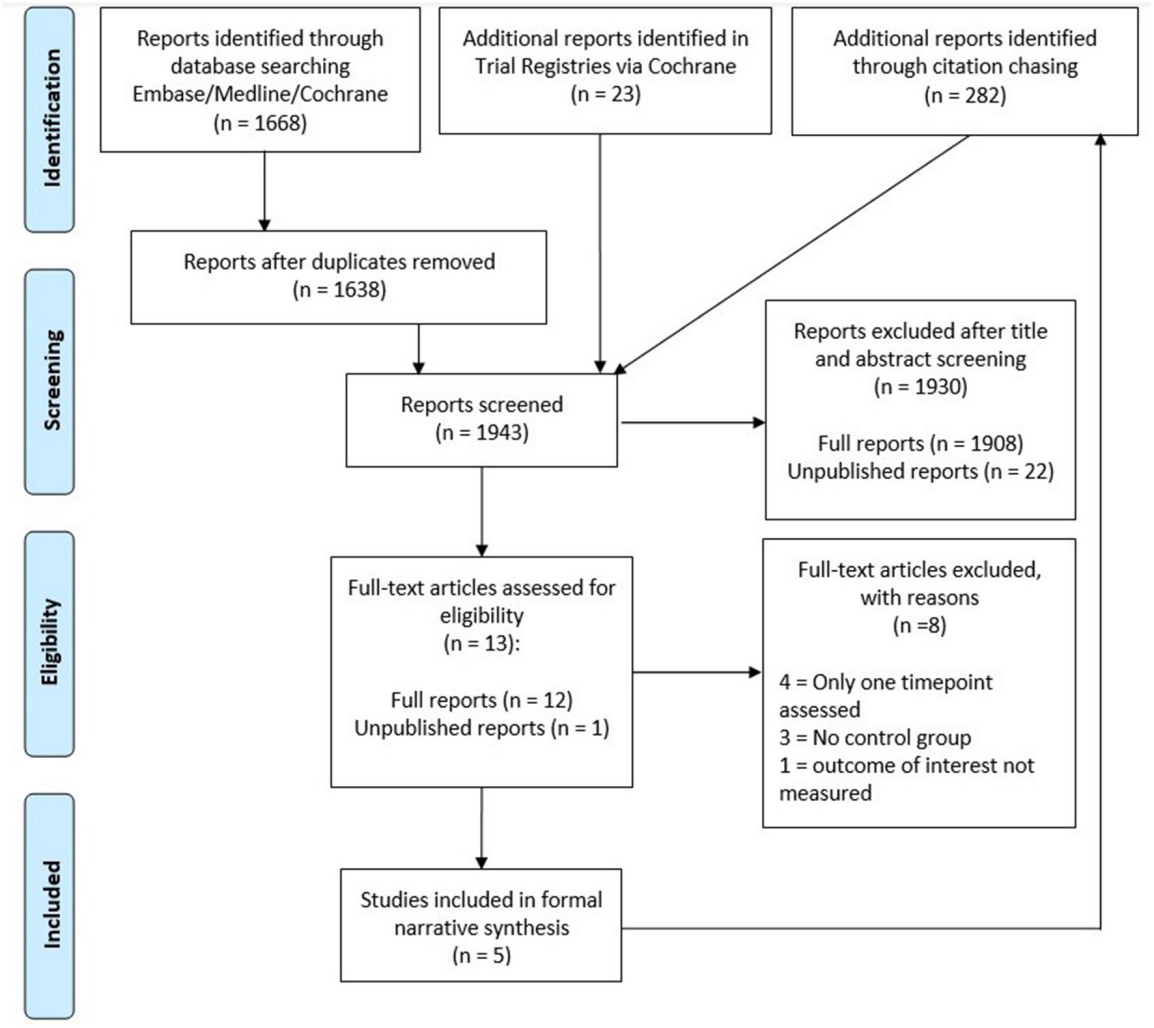
Prisma flow diagram detailing search results and screening process.

An overview of the article selection process can be found in Figure 2. In total, our search retrieved 1,668 results from Cochrane and Ovid databases, with 1,638 results remaining after deduplication. A further 23 results were identified in trial registries.

JH screened the titles and abstracts to identify potentially eligible studies, excluding 1,908 published and 18 unpublished reports in the process. Furthermore, we were unable to obtain data from 4 of the remaining unpublished reports. As a result, 12 published studies and 1 unpublished study were included in the full text screening. JH and CK performed the full text screening independently. Of these 13 studies, we excluded 3 studies without a second sampling timepoint, 4 studies without an appropriate control group and 1 study that did not assess the outcomes of interest. Study inclusion was decided in mutual discussion and in consultation with WA when necessary. Forward and backward citation chasing was performed for the 5 included studies on Scopus and identified a further 282 results. These were screened by JH, but no further eligible studies were identified by this method.

### Critical Appraisal of the Studies Revealed Heterogeneity Among the Studies in Terms of Populations, Treatment and Laboratory Methods Employed

Data from the 5 included studies was extracted by two authors (JH, CK) independently using a standardized table. The quality was assessed independently by JH and CK according to the Newcastle Ottawa Scale (NOS) for observational studies and the revised Cochrane risk-of-bias tool (ROB 2.0) for randomized studies. Any disagreements were resolved by mutual discussion with involvement of WA where necessary. Corresponding authors of any studies with missing data were contacted by e-mail twice to request clarification.

The included studies are summarized in Table 1. Of the five included studies, two focused on patients with COPD (26, 27), two focused on patients suffering from asthma (28, 29), and one focused on patients with chronic rhinosinusitis (30). Two studies were randomized-controlled trials (26, 28). The remaining three studies were designed as cohort studies (27, 30), one of them nested in a randomized controlled trial (29). One study took place in Italy (26), one in the United Kingdom (27), while the remaining three were conducted in the United States of America (28–30). One study investigated the effect of oral intake of prednisone (30 mg/day) (27), one study investigated the effect of topical nasal application of mometasone furoate monohydrate (200 μg/day) (30), and the remaining three studies investigated the effect of fluticasone propionate (FP) inhalation in varying dosages (26, 28, 29). Study design was considerably heterogenous, the sample sizes ranging from 5 patients (30) to 230 (27), and the duration of treatment with CS ranging from 14 days (27) to 12 months (26). Only one of the included studies assessed the microbiome changes with mNGS (29), three studies used 16S sequencing, and one study used a commercially available 16S rRNA qPCR assay (26). The DNA extraction kits and sequencing platforms varied between the studies (see Table 1).

**Table 1:** Summary of included studies and of reported microbiota changes.

**References**	**Patients**	**Intervention/Control**	**Methods**	**Outcomes**			**Study design**
				**Diversity**	**Composition**	**Burden**	
Contoli et al. (26)	*n* = 60, steroid-naïve COPD patients with stable disease	**Steroids:** (*n* = 30) FP 500 μg for 12 months, inhaled twice daily (total/day: 1000 μg), co-intervention: salmeterol 50 μg, **Controls:** (*n* = 30) only salmeterol 50 μg	**qPCR assays**: multiplex PCR assay RespiFinder RG® (Qiagen); Microbial DNA qPCR Arrays BAID-1404ZRR-24 Respiratory Infections (Qiagen).	**Steroids:** α diversity (restricted to 41 species) was significantly increased after 1 year **Controls:** No significant change	**Steroids:** significant increase of the *Firmicutes* phylum and significant decrease in *Proteobacteria* phylum. Significantly increased relative abundance in *Streptococcus pneumoniae* and *Haemophilus influenzae* after 1 year when compared to controls. **Controls:** at baseline, significantly increased relative abundance of *Prevotella spp*. (p < 0.05) when compared to treatment samples.	Assessed by culture **Steroids:** bacterial load significantly higher after 1 year **Controls:** no change	Randomized, open-label, blinded endpoint study, Italy, 12 months
Durack et al. (28)	*n* = 42, atopic asthmatic subjects	**Steroids:** (*n* = 28) FP 250 μg for 6 weeks, inhaled twice daily (total/day: 500 μg), **Controls:** (*n* = 14) placebo inhaled twice daily	**16S PCR**; DNA extraction: AllPrep kit (Qiagen); 16S PCR on V4 region; qPCR with universal primers to assess burden; sequencing: Illumina MiSeq	No significant changes in either group	Steroids: subgroup of **steroid-responders** (*n*=8): increase in Microbacteriaceae and *Neisseria/Moraxella* species, decrease in *Fusobacterium* and *Dialister* **Control:** (*n* = 8) increase in *Eikenella* and *Mycoplasmataceae*, decrease in *Prevotellaceae*	No significant changes in either group	Randomized-controlled trial (RCT), United States, study duration not specified
Ramakrishnan et al. (30)	*n* = 5, 4 adult males with chronic non-infectious rhinitis and 1 healthy control	**Steroids:** (*n* = 4) mometasone furoate monohydrate 100 μg (50 μg twice daily in each nostril) for 4 weeks as nasal spray twice daily (total/day: 200 μg), **control:** (*n* = 1) mupirocin twice daily	**16S PCR**; 16S PCR on V1-V3 regions; sequencing: 454/Roche Life Sciences GS-FLX instrument using Titanium chemistry (Roche Life Sciences)	**Steroids:** transient upwards trend in 2 patients, no significant change overall **Control:** downward trend	**Steroids:** persistent non-significant increase in relative abundance in the genera *Corynebacteria* and *Gordonia* after treatment. Transient increase in *Staphylococcus* and decrease in *Moraxella* and *Streptococci*. **mupirocin:** effects were not evident along principal component axes 1 and 2, indicating a different response than to steroids	**Steroids:** no change **Control:** 50% of PCRs after treatment did not yield sequences, indicating a bactericidal effect	Prospective pilot cohort study, United States, 12 weeks
Turturice et al. (29)	*n* = 19, young adult, atopic asthmatics and age-matched controls	**Steroids:** (*n* = 13) FP 100 or 500 μg for 7 weeks, inhaled twice daily (total/day: 200 or 1,000 μg), co-intervention: salbutamol 100 μg as needed **Healthy controls:** (*n* = 6) no treatment	**mNGS**; DNA extraction: QIAamp Virus Spin Minelute kit (Qiagen) sequencing: Illumina MiSeq using the v3-600 kit for 301 paired-end read length	**Steroids:** significant increase in α diversity **Controls:** result not provided	**Steroids:** Asthma Phenotype 1 (phenotype identified by study through unsupervised clustering of chemo- and cytokines): significant reduction of *E. faecium* and *E. faecalis* Asthma Phenotype 2 with decreased baseline pulmonary function and increased obstruction: significant reduction of *S. pneumoniae* and *Neisseria meningitidis* **Controls:** result not provided	Not assessed	Nested substudy of RCT with healthy control cohort, United States, 8–11 weeks
Wang et al. (27)	*n* = 94 exacerbation events from 87 COPD patients	**Steroids:** (*n* = 73) prednisone 30 mg for 14 days, per os once daily, co-intervention (only for *n*= 65): antibiotics (same as controls) **Controls:** (*n* = 21) antibiotics (amoxicillin or doxycycline) for 7 days per os	**16S PCR**; DNA extraction: Qiagen DNA Mini kit (Qiagen); 16S PCR on V3-V5 regions; Sequencing: 454 Genome Sequencer FLX platform (454 Life Sciences; Roche Diagnostics)	**Steroids only:** trend to decrease in Shannon's H **Antibiotics** **±** **steroids**: trend to increase in Shannon's H	**Steroids only:** trend (non-significant) increase of Proteobacteria, decrease of Firmicutes. On genus level: decrease of *Streptococcus* and increase of *Haemophilus* and *Moraxella* **Antibiotics** **±** **steroids**: trend (non-significant) increase of *Firmicutes*, decrease of *Proteobacteria*. On genus level: increase of *Streptococcus*, decrease of *Haemophilus*. Significant decrease of *Moraxella*.	Not assessed	Longitudinal prospective cohort study, United Kingdom, 12 months

### Although the Studies Are Heterogeneous and, in Some Cases, Small, the Outcome Results Suggest That Steroid Therapy Affects the Respiratory Microbiome

Regarding the results on the **diversity** of the respiratory microbiome, two studies detected significant increases in the respiratory microbiome diversity of patients treated with CS (26, 29) and one study including 5 participants identified a transient increase in diversity in 2 of the participants treated with CS (30). Durack et al. did not find any significant change in the diversity after CS treatment (28) and Wang et al. found a trend to a decrease in diversity (27). Overall, 2 studies showed significant increases, whereas the remaining studies found no significant changes.

**Changes in the composition** of the microbiome were observed in all the studies. These were significant in 3 studies (26, 28, 29). Two studies detected a shift in the *Firmicutes* to *Proteobacteria* ratio (26, 27). Contoli et al. found a significant increase in *Firmicutes* paralleled by a significant decrease in *Proteobacteria* (26), while Wang et al. observed a non-significant trend in the opposite direction (27). On the genus and species level a variety of compositional shifts were detected. Contoli et al. observed increased relative abundance of *S. pneumoniae* and *H. influenzae* in COPD patients following treatment with fluticasone (26). Turturice et al. observed a decrease in *S. pneumoniae* and *N. meningitidis* in one of the asthma phenotypes following treatment with fluticasone (29). An increase in *Microbacteriaceae, Neisseria* and *Moraxella* was observed by Durack et al. in asthma responders (28). An overview of all bacterial taxa listed in the included studies and mentioned in this manuscript can be found in Table 2.

**Table 2:** Bacterial Glossary of recognized upper airway taxa.

**Phylum**	**Family**	**Genus**	**Species**
Actinobacteria	Microbacteriaceae*		
	Corynebacteriaceae	**Corynebacterium**	
	Nocardiaceae	**Gordonia**	
Firmicutes*	Staphylococcaceae	**Staphylococcus**	
	*Streptococcaceae*	*Streptococcus*	pneumoniae*
	Enterococcaceae	Enterococcus	faecium*
			faecalis*
	Veillonellaceae	Dialister*	
Fusobacteria	Fusobacteriaceae	Fusobacterium*	
Proteobacteria*	Neisseriaceae	Neisseria*	meningitidis
		Eikenella*	
	Moraxellaceae	Moraxella*	
	Pasteurellaceae	**Haemophilus**	influenzae*

In bold with asterisk*: families, genera and species with significant increases in abundance were detected after CS. **In bold without asterisk**: non-significant increases after CS. Underlined with asterisk*: significant decreases after CS. Underlined without an asterisk: non-significant decreases after CS.

*Where the included studies provided conflicting results, only the significant result was used for the table*.

Only three studies assessed the total **bacterial burden** of the airway microbiome (26, 28, 30) and only two of these employed culture-independent methods for the assessment. Only one study found a significant change (an increase) in total bacterial burden and this study employed culture to assess this parameter (26).

**Figure 3:**
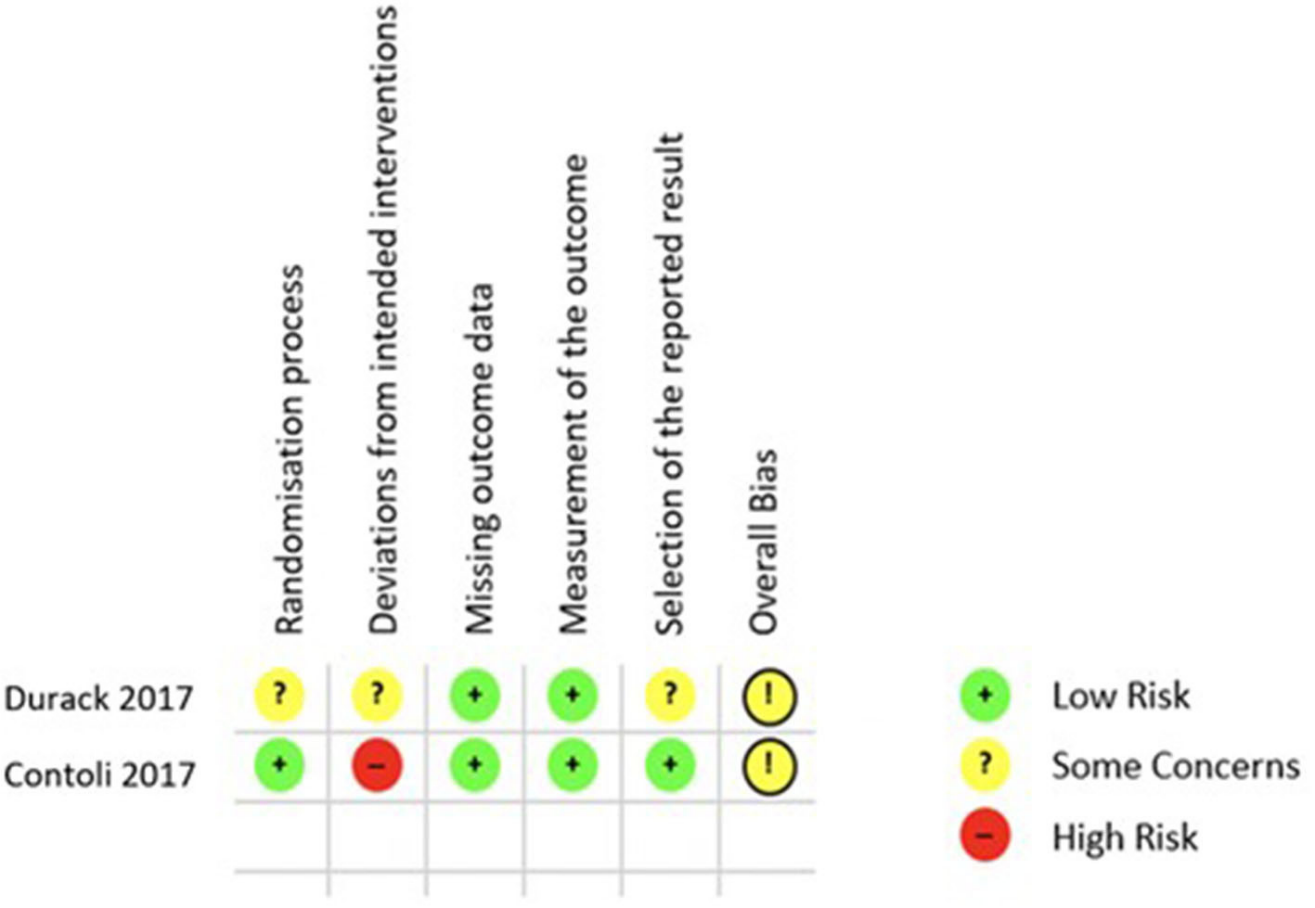
**ROB 2.0 results** quality appraisal of RCTs. ROB 2.0 results of the two included RCTs, both with the result of some concerns in the overall bias.

**Figure 4:**
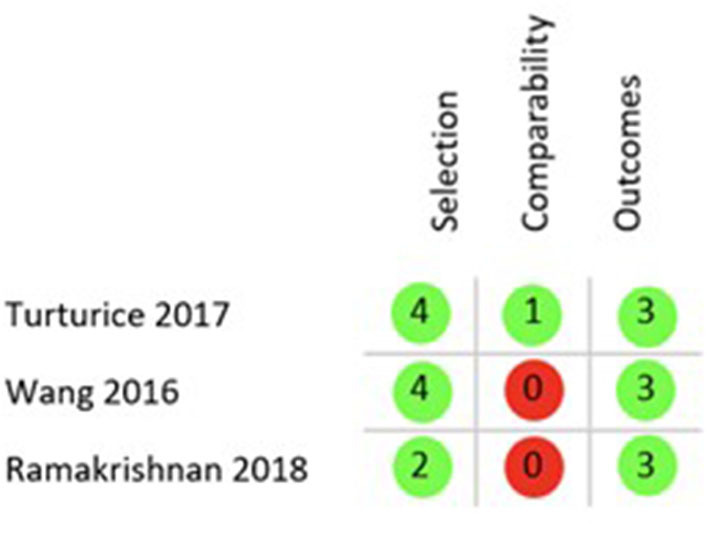
**NOS results** quality appraisal of observational studies. NOS results of the three included cohort studies, two with an overall poor rating due to the lack of comparability between cohorts and one with a good rating.

Finally, the quality of the 5 included studies was appraised with two different tools, ROB 2.0 for RCTs and the Newcastle Ottawa Scale (NOS) for cohort studies. Applying the ROB 2.0 both included RCTs were judged to have some concerns of overall bias as is shown in Figure 3 (26, 28). Using the NOS as described in the Methods section only one of the cohort studies was of good quality regarding risk of bias (29), while the remaining two both rank as poor quality as shown in Figure 4 (27, 30).

## Discussion

Although there was considerable heterogeneity among the few available studies, treatment with CS appeared to have a significant impact on the makeup of the microbiome.

The overall trend for microbiome diversity following treatment with CS was an increase: two studies showed a significant increase in α-diversity after inhalation of FP (26, 29). One of these studies included patients with stable COPD who received 12 months of FP, the other young adult asthmatics who received FP for 6 weeks. Perhaps this can be interpreted as an indication of an effect independent of the underlying disease, which starts relatively soon and extends for a prolonged period. An increased diversity appears to be beneficial in COPD and protective against asthma (31, 32). The only study with an opposite, albeit non-significant, trend for a decreased diversity was the only study which used oral CS for 14 days (27). Unfortunately, due to the limited number of studies it is impossible to conclude whether this difference was related to the route of CS application.

Most studies detected significant shifts in the composition of the airway microbiome (26, 28, 29). An overarching trend regarding the effect of corticosteroid treatments was detected despite the heterogeneity of the study designs and populations, whereas the observed heterogeneity limited further conclusions regarding the direction of the effects. In the respiratory microbiome, *Proteobacteria* and *Firmicutes* appeared to be inversely correlated (31). Additionally, elevated levels of the *Proteobacteria* phylum in COPD appeared to be associated with exacerbations (pre-treatment) (27, 33). Contoli et al. found the *Proteobacteria* phylum to be significantly reduced in the group treated with FP for 12 months, perhaps indicating a beneficial effect on microbiome composition (26). Contrarily, Durack et al. found *Neisseria* and *Moraxella* (both belonging to the *Proteobacteria* phylum, see Table 2) to be increased in their cohort of steroid-responders (28). Only one study found a significant effect of steroid treatment on the burden of the microbiome in COPD patients (26). This specific endpoint was measured using culture (not a culture-independent method), suggesting a possible methods effect.

Baseline assessments of steroid-naïve asthma patients by Durack et al. tended to have a higher phylogenetic diversity (Faith index) compared to healthy controls (*p* = 0.06) (28). A further study comparing steroid-naïve asthma patients to healthy controls found no difference in α-diversity, but detected a significant composition difference on the taxonomical level (34). A clear differentiation between the influence of the treatment and that of the underlying condition is difficult to achieve, particularly in such small cohorts.

Garcia-Nunez et al. found a significant correlation between lung function and bacterial diversity in sputum in COPD patients (31), however, whether these differences also stem from the underlying disease or the treatments prescribed is not known. In COPD, inhaled steroids are an important component of the available armamentarium and significantly slow the decline in quality of life and lower the exacerbation rate (35). Inhaled CS treatment is likewise a pillar of asthma therapy recommended by national and international guidelines (36, 37). The role of the respiratory microbiome in mediating these effects is only being uncovered gradually. Significant differences in the composition of the microbiome were detected between ICS-responders and non-responders (28, 38), potentially allowing for better prediction of treatment response or the development of new treatment options.

Additionally, it can be presumed that the route of application (inhaled or systemic) and substance choice might also play a significant role in how CS affect the microbiome. Due to the current paucity of studies, our review unfortunately cannot adequately assess this question. Three of the included studies investigated the effect of FP on the respiratory microbiome (26, 28, 29). FP might, however, be an outlier among inhaled steroid therapies, as it has been associated with an increased risk of pneumonia in adults as discussed in an overview of systematic reviews comparing FP with budenoside by Janson et al. (39). The authors listed differences in pharmacokinetics and immunosuppressive efficacy as potential reasons for this difference in pneumonia incidence (39). Other pharmacological treatments may also influence the airway microbiome. For example, Durack et al. proposed that the compositional shifts observed in the placebo cohort of their study may be caused by the lactose contained in the placebo medication (28).

Eosinophilia appears to further influence the lung microbiome parameters in COPD and asthma (26, 27, 40). Additional factors such as diet and probiotics also affect the makeup of the respiratory microbiome via the gut-lung axis (13, 15, 41). However, these factors were not adequately assessed or controlled for in the studies identified by and included in our systematic review.

The main limitation of this systematic review is the small number of studies fulfilling our inclusion criteria. Three studies covering the topic of interest only analyzed samples from one time point (38, 42, 43). The teams of two further studies responded to our requests for further information but did not include data from non-steroid treated controls (33, 44). The resulting small number of selected studies and their recency is certainly related to the relative novelty of this field of microbiome research. As evidence of ongoing exploration, we identified and contacted the authors of four registered clinical trials and one conference abstract without a full published report investigating this topic. However, only one team had already finalized data analysis by that point. Thus, an update of this systematic review including the data from these studies would be interesting once these ongoing studies become available.

The second apparent limitation is the great heterogeneity between the five included studies regarding analytic methods, such as sequencing techniques and platform, study populations, tested CS agents, dosages, applications and duration of CS treatment. The control populations varied between healthy controls (29, 30), patients with the same disease receiving placebo (28) or SOC (26, 27). Each of these factors potentially affects the outcomes of interest, complicating the interpretation and comparison between the studies. As an example, the comparability of asthma patients who manage as SOC with a pure bronchodilator treatment is limited because this control group is closer to a healthy population. On the other hand, it would be difficult to separate the effect of corticosteroids differently. Consequently, the discrepancies in the reported outcomes may also in part be due to the different methods used, particularly between studies using culture vs. non culture-based methods. Therefore, it is challenging to disentangle the true CS effect from these or other unidentified confounding factors on microbiome diversity, burden and composition.

Each of the five included studies also has its own limitations. In particular the study by Ramakrishnan et al. (30) is limited by the very small number of participants but nevertheless fulfilled the inclusion criteria of this systematic review. Similarly, the studies by Durack et al. (28) and Turturice (29) are limited by their small sample sizes. But it is of note that the study by Turturice et al. was the only observational study to score well in the quality appraisal process (29). Contoli et al. (26) showed a very well-structured study design with a sufficient sample size. However, the use of the qPCR assay does not allow a full assessment of the respiratory microbiota by limiting the number of detectable species. The observational study design by Wang et al. (27) increases the amount of bias and limits the interpretability of the results. In the quality appraisal both included RCTs were judged to have some concerns of overall bias as is shown in Figure 3 (26, 28).

The strengths of this review include the methodologically precise execution and the maximization of the scope of the search. This allowed an accurate documentation of the current status quo of research on this question and the considerable heterogeneity of methodology. Thus, our results importantly show the limits of the current understanding of this important topic and and may substantially inform the planning of future studies in this field.

## Conclusion

The identified studies showed CS to significantly affect the composition and possibly the diversity of the respiratory microbiome. However, there was relevant disagreement regarding the nature of these effects and the direction of the changes, and the currently available data did not allow the drawing of clear conclusions as to the cause of these partially discrepant results. CS are frequently used in medicine and the relevant effects of the microbiome on health and disease are increasingly recognized. Therefore, there is an urgent need to better understand the true and various effects of systemic and inhaled CS on the respiratory microbiome in different diseases. This could facilitate a more targeted use of CS.

## Data Availability Statement

The raw data supporting the conclusions of this article will be made available by the authors, without undue reservation.

## Author Contributions

JH formulated the research question, planned, and performed the systematic literature search and selection process and was a major contributor in writing the manuscript. WA provided guidance in formulating the research question and data interpretation and was a major contributor to the manuscript. MD provided feedback on the selected studies regarding methodology and on the manuscript. CK provided guidance in formulating the research question, participated in the study selection and quality appraisal, assisted with data interpretation and was a major contributor to the manuscript. JH, MD, WA, and CK contributed substantially to the data interpretation, and the writing of the manuscript. All authors contributed to the article and approved the submitted version.

## Conflict of Interest

The authors declare that the research was conducted in the absence of any commercial or financial relationships that could be construed as a potential conflict of interest.

## Abbreviations

CS: corticosteroids
FMT: fecal microbiota transfer
FP: fluticasone propionate
ICS: inhaled corticosteroids
mNGS: metagenomic next generation sequencing
NOS: Newcastle Ottawa Scale
RCT: randomized controlled trial
ROB 2.0: revised Cochrane risk-of-bias tool
SOC: standard of care.
