# Supplementary material for: The Effects of Corticosteroids on the Respiratory Microbiome: A Systematic Review
Source: Front Med (Lausanne). 2021 Mar 10;8:588584. doi: 10.3389/fmed.2021.588584 (PMC7988087; doi:10.3389/fmed.2021.588584)
Supplement: Supplementary file 1 [file Data_Sheet_1.docx]

**Table 3: Full Search String Ovid**

Search Results Medline and Embase via Ovid 05.06.2019

| - 1. | - exp respiratory system/ or exp respiratory tract disease/ | - 4089387 |
| --- | --- | --- |
| - 2. | (asthma* or lung* or pulmo* or respiratory or COPD or (chronic adj3 airflow adj3 (obstruction or disease* or disorder*)) or painful breathing or (sputum adj3 discoloration) or diaphragm or mediastinum or tracheobronchomalacia or bronchopneumonia or tracheobronchomegaly or ciliary motility disorders or kartagener syndrome or (vocal cord adj3 (dysfunction or paralysis)) or voice disorders or acute chest syndrome or alpha 1-antitrypsin deficiency or cystic fibrosis or hemoptysis or hepatopulmonary syndrome or tuberculosis or epistaxis or rhinitis or rhinoscleroma or pleura* or chylothorax or empyema or hemopneumothorax or hemothorax or hydropneumothorax or hydrothorax or pneumothorax or altitude sickness or apnea or cough or dyspnea or hoarseness or hyperventilation or meconium aspiration syndrome or mouth breathing or sarcoglycanopath* or tachypnea or ((alveolitis or aspergillosis) adj3 allergic) or common cold or influenza or legionellosis or pleurisy or pneumonia or sinus* or supraglottitis or choanal atresia or tracheitis or cough or bronch* or trachea* or airway* or nasopharyn* or oropharyn* or epipharyn* or rhinopharyn* or laryn* or pharyn* or nose or nasal or apparatus respirator* or systema respiratorium or (respiration adj3 (apparat* or arch or track or tract or system))).ti,ab. | 5043520 |
| - 3 | 1 or 2 | 6137532 |
| - 4 | - exp glucocorticoid/ or steroid/ or steroid hormone/ or corticosteroid/ or exp adrenal cortex hormones/ | 1376979 |
| - 5 | (glucocorticoid* or glucocorticoidsteroid* or glucocorticosteroid* or glucocortoid* or glycocorticoid* or glycocorticosteroid* or corticosteroid* or steroid* or alclometason* or algeston* or amcinonid* or amelometason* or beclometason* or betamethasone* or budesonide* or butixocort* or chloroprednison* or ciclesonid* or ciprocinonid* or clobetasol* or clobetason* or clocortolon* or cloprednol* or cortison* or cortisol* or cortivazol* or deflazacort* or dexamethasone* or diflorason* or diflucortolone* or difluprednate* or domoprednate* or drocinonide* or dutimelan* or etiprednol dicloacetate or fluclorolone* or fludrocortisone* or fludroxycortid* or flumetason* or flumoxonide* or flunisolide* or fluocinolon* or fluocinonide* or fluocortin* or fluocortolon* or fluorometholon* or flupredniden* or fluprednisolon* or fluticasone* or formocortal* or mometasone furoate or halcinonide* or halometasone* or halopredon* or hydrocortisone* or icometasone enbutate or isoflupredon* or itrocinonide* or locicortolone dicibate or lorinden or loteprednol* or mazipredon* or medryson* or meprednison* or nicocortonide* or nivacortol* or oropivalon* or paramethason* or prednisolon* or prednisone* or pregnenolon* or procinonide* or promestriene* or resocortol* or rimexolon* or rofleponide* or ticabesone* or timobeson* or tipredane* or tixocortol* or triamcinolon* or ulobetasol propionate or uniderm* or vamorolon* or zoticason*).ti,ab. | 1155776 |
| - 6 | 4 or 5 | 1838287 |
| - 7 | microbiota/ or bacterial flora/ or microbiome/ or 16S Ribosomal RNA/ or metagenomics/ or whole genome sequencing/ or DNA barcoding/ or DNA barcoding, taxonomic/ | 165040 |
| - 8 | (ecogenomic* or metagenomic or genomic* or (Microbial adj3 (Composition or Structure)) or microbiome* or microbiota or micro-biota or micro-biome* or microbe* or microflora or bacterial flora or microbial flora or 16s ribosomal gene or 16s ribosomal rna or 16s rna or 16S rRNA or 16SrRNA or ribonucleic acid 16s or ribosomal 16S RNA or ribosomal rna 16s or rna, ribosomal, 16s or rrna 16s or ((DNA or molecular) adj3 (barcod* or bar-cod* or bar cod*) adj3 taxonomic) or (genome adj4 sequencing)).ti,ab. | 921952 |
| - 9 | 7 or 8 | - 964420 |
| - 10 | 3 and 6 and 9 | 2158 |
| - 11 | remove duplicates from 10 | 1752 |
| - 12 | 11 not (exp animal/ not human/) | 1610 |
