# Supplementary material for: The Effects of Corticosteroids on the Respiratory Microbiome: A Systematic Review
Source: Front Med (Lausanne). 2021 Mar 10;8:588584. doi: 10.3389/fmed.2021.588584 (PMC7988087; doi:10.3389/fmed.2021.588584)
Supplement: Supplementary file 2 [file Data_Sheet_2.docx]

**Table 4: Full Search String Cochrane**

Cochrane Search 10.06.2019

| #1 | (asthma* or lung* or pulmo* or respiratory or COPD or (chronic NEAR/3 airflow NEAR/3 (obstruction or disease* or disorder*)) or ‘painful breathing’ or (sputum NEAR/3 discoloration) or diaphragm or mediastinum or tracheobronchomalacia or bronchopneumonia or tracheobronchomegaly or ‘ciliary motility disorders’ or ‘kartagener syndrome’ or ((‘vocal cord’ NEAR/3 (dysfunction or paralysis)) or ‘voice disorders’ or ‘acute chest syndrome’ or ‘cystic fibrosis’ or 'alpha 1 antitrypsin syndrome' or hemoptysis or ‘hepatopulmonary syndrome’ or tuberculosis or epistaxis or rhinitis or rhinoscleroma or pleura* or chylothorax or empyema or hemopneumothorax or hemothorax or hydropneumothorax or hydrothorax or pneumothorax or ‘altitude sickness’ or apnea or cough or dyspnea or hoarseness or hyperventilation or ‘meconium aspiration syndrome’ or ‘mouth breathing’ or sarcoglycanopath* or tachypnea or ((alveolitis or aspergillosis) NEAR/3 allergic) or ‘common cold’ or influenza or legionellosis or pleurisy or pneumonia or sinus* or supraglottitis or ‘choanal atresia’ or tracheitis or cough or bronch* or trachea* or airway* or nasopharyn* or oropharyn* or epipharyn* or rhinopharyn* or laryn* or pharyn* or nose or nasal or 'apparatus respirator*' or 'systema respiratorium' or (respiration NEAR/3 (apparat* or arch or track or tract or system)))):ti,ab,kw | 226486 |
| --- | --- | --- |
| #2 | (glucocorticoid* or glucocorticoidsteroid* or glucocorticosteroid* or glucocortoid* or glycocorticoid* or glycocorticosteroid* or corticosteroid* or steroid* or alclometason* or algeston* or amcinonid* or amelometason* or beclometason* or betamethasone* or budesonide* or butixocort* or chloroprednison* or ciclesonid* or ciprocinonid* or clobetasol* or clobetason* or clocortolon* or cloprednol* or cortison* or cortisol* or cortivazol* or deflazacort* or dexamethasone* or diflorason* or diflucortolone* or difluprednate* or domoprednate* or drocinonide* or dutimelan* or etiprednol dicloacetate or fluclorolone* or fludrocortisone* or fludroxycortid* or flumetason* or flumoxonide* or flunisolide* or fluocinolon* or fluocinonide* or fluocortin* or fluocortolon* or fluorometholon* or flupredniden* or fluprednisolon* or fluticasone* or formocortal* or mometasone furoate or halcinonide* or halometasone* or halopredon* or hydrocortisone* or icometasone enbutate or isoflupredon* or itrocinonide* or locicortolone dicibate or lorinden or loteprednol* or mazipredon* or medryson* or meprednison* or nicocortonide* or nivacortol* or oropivalon* or paramethason* or prednisolon* or prednisone* or pregnenolon* or procinonide* or promestriene* or resocortol* or rimexolon* or rofleponide* or ticabesone* or timobeson* or tipredane* or tixocortol* or triamcinolon* or ulobetasol propionate or uniderm* or vamorolon* or zoticason*):ti,ab,kw | 83985 |
| #3 | (ecogenomic* or metagenomic or genomic* or (Microbial NEAR/3 (Composition or Structure)) or microbiome* or microbiota or micro-biota or micro-biome* or microbe* or microflora or 'bacterial flora' or 'microbial flora' or '16s ribosomal gene' or '16s ribosomal rna' or '16s rna' or '16S rRNA' or 16SrRNA or 'ribonucleic acid 16s' or 'ribosomal 16S RNA' or 'ribosomal rna 16s' or 'rna, ribosomal, 16s' or 'rrna 16s' or ((DNA or molecular) NEAR/3 (barcod* or bar-cod* or ‘bar cod*’) NEAR/3 (taxonomic)) or (genome NEAR/4 sequencing)):ti,ab,kw | 9486 |
| #4 | #1 AND #2 AND #3 | 81 (58 reports, 23 trials) |
